# The Effects of Methadone and Buprenorphine on Testis of Rats

**DOI:** 10.5812/ijhrba.6861

**Published:** 2012-11-26

**Authors:** Karen F. Corsi

**Affiliations:** 1 Division of Substance Dependence, University of Colorado School of Medicine, Colorado, United States

**Keywords:** Buprenorphine, Methadone, Testis

*Dear Editor,*

I am writing in response to the article, “A quantitative and qualitative study of rat testis following administration of methadone and buprenorphine” by Zahra Heidari, Hamidreza Mahmoudzadeh-Sagheb & Farhad Kohan in Volume 1(1): 14-17, 2012 (1).

This article is addresses an important subject, that of hypogonadism and other changes in seminiferous tubules and testis after administration of methadone or buprenorphine in rats. The subject is important given that opiate use itself has been shown to cause low FT levels in men and therefore should be examined systematically to see how to prevent hormonal issues for drug-using men (2). The authors do not note any quantitative differences between groups on testis volume in this study however, indicating that possibly the dosage of methadone and buprenorphine given were not sufficient to produce those changes. Conversely, histopathology did show qualitative changes, which are reported but not qualified as significant or not. It is unclear what the measures of significance for these changes in rat’s testis are. The figures presented show that there are changes, it is not easily distinguishable as a significant clinical outcome. The authors say that “previous studies showed that both methadone- and buprenorphine-treated men had lower free testosterone, LH, and estradiol levels than those in the age-matched control groups”. However, there is no reference given to assess what type of study this was. It would be necessary to know if the levels of opiate use were controlled for and how this was performed, as this could also be a cause for differences in testis measures.

Overall, this is a relevant topic and well-executed study indicating preliminary trends toward the benefit of using buprenorphine over methadone for young men. However, more precise measures and description of histopathologic changes would strengthen this argument. This study adds to the literature on this topic regarding the effects of methadone and buprenorphine on gonadal levels in men. Due to conflicting research outcomes that exist on this topic (as pointed out here by the authors), more studies such as this one are needed to determine clinical differences between opioid treatments.
